# Methodological quality for systematic reviews of adverse events with surgical interventions: a cross-sectional survey

**DOI:** 10.1186/s12874-021-01423-6

**Published:** 2021-10-25

**Authors:** Xiaoqin Zhou, Linji Li, Lifeng Lin, Ke Ju, Joey S. W. Kwong, Chang Xu

**Affiliations:** 1 Department of Clinical Research Management, West China Hospital, Sichuan University, Chengdu, China; 2 Department of Anesthesiology, Nanchong Central Hospital, The Second Clinical Medical College, North Sichuan Medical College, Nanchong, China; 3 Department of Anesthesiology, West China Hospital, Sichuan University, Chengdu, China; 4 Department of Statistics, Florida State University, Tallahassee, FL USA; 5 West China School of Public Health and West China Fourth Hospital, Sichuan University, Chengdu, China; 6 Global Health Nursing, St. Luke’s International University, Tokyo, Japan; 7 Department of Population Medicine, College of Medicine, Qatar University, Al Jamiaa Street, P. O. Box 2713, Doha, Qatar

**Keywords:** Adverse event, Methodological quality, Surgical intervention, Systematic review

## Abstract

**Background:**

An increasing number of systematic reviews assessed the safety of surgical interventions over time. How well these systematic reviews were designed and conducted determines the reliability of evidence. In this study, we aimed to assess the methodological quality of systematic reviews on the safety of surgical interventions.

**Methods:**

We searched PubMed for systematic reviews of surgical interventions with safety as the exclusive outcome from 1st-Jan, 2015 to 1st-Jan, 2020. The methodological quality of eligible systematic reviews was evaluated according to the AMSTAR 2.0 instrument. The primary outcomes were the number of methodological weaknesses and the global methodological quality. The proportion of each methodological weakness among eligible systematic reviews was compared by three pre-defined stratification variables. The absolute difference of the proportion (PD) was used as the effect estimator, with the two-tailed z-test for the significance.

**Results:**

We identified 127 systematic reviews from 18,636 records. None (*n* = 0, 0.00%) of them could be rated as “high” in terms of the global methodological quality; in contrast, they were either rated as “low” (*n* = 18, 14.17%) or as “critically low” (*n* = 109, 85.83%). The median number of methodological weaknesses of these systematic reviews was 8 (interquartile range, IQR: 6 to 9), in which 4 (IQR: 2 to 4) were critical weaknesses. Systematic reviews that used any reporting guideline (e.g., domain 13, PD = -0.22, 95% CI: − 0.39, − 0.06; *p* = 0.01) and developed a protocol in advance (e.g., domain 6, PD = -0.20, 95% CI: − 0.39, − 0.01; *p* = 0.04) were less likely to have methodological weakness in some domains but not for the rest (e.g., domain 8, PD = 0.04, 95% CI: − 0.14, 0.21; *p* = 0.68; with protocol vs. without).

**Conclusions:**

The methodological quality of current systematic reviews of adverse events with surgical interventions was poor. Further efforts, for example, encouraging researchers to develop a protocol in advance, are needed to enhance the methodological quality of these systematic reviews.

**Supplementary Information:**

The online version contains supplementary material available at 10.1186/s12874-021-01423-6.

## Introduction

Systematic reviews and meta-analyses have been increasingly popular in healthcare intervention comparisons that offer an important source of evidence to support the decision-making [1, 2]. By quantitatively or qualitatively synthesizing all available findings, systematic reviews and meta-analyses are expected to provide the most comprehensive and informative evidence for employing optimal healthcare interventions [3]. In the hierarchy of evidence pyramid, qualified systematic reviews and meta-analyses are ranked at the top over other sources of evidence [4, 5].

In healthcare intervention, effectiveness and safety are the two main outcomes of concerns by clinicians, but the former frequently draws more attention over the latter. This preference has been reflected in the related publications; most systematic reviews and meta-analyses were about effectiveness rather than safety [6]. It is arguably reasonable to some extent because patients with serious symptoms may care more about the pain caused by disease rather than potential adverse events, and for many interventions, adverse events are rare [7]. As a type of invasive operation, surgical interventions are more likely to cause adverse events, especially serious adverse events, than drug or other interventions [8]. In addition, the prognosis of a patient is strongly associated with the experience and proficiency of the surgeon. The safety of surgical interventions, therefore, raised more concerns.

An adverse event is any harmful medical occurrence in a patient in clinical practice; it could be an adverse effect, adverse reaction, harm, toxicity, or complication during healthcare interventions [9, 10]. Some serious adverse events of surgical interventions have been frequently reported in previous studies, such as postoperative hemorrhage, septic shock, or some site-specific adverse events (e.g., pulmonary embolism) [11–20]. The assessment of safety is more complex than effectiveness because the safety outcome is generally less common and thus faces the problems of underpowered analyses and possibly zero-events [21, 22]. These make the inference of adverse events more susceptible to random and systematic errors (e.g., selection bias); whether appropriate methods were applied would largely impact the credibility of the results [23].

An increasing number of systematic reviews and meta-analyses of safety for surgical interventions have been published, but it is unclear whether they were designed and conducted well. Adie et al. [24] have investigated the quality of meta-analyses of surgical interventions and suggested poor compliance with methodology recommendations. Although the study did not specify whether the effectiveness or safety was focused, it indicated that serious concerns should be taken on the methodological quality of systematic reviews of surgical interventions. We hypothesized that systematic reviews of surgical interventions on adverse events might face the same problem. In this article, we investigated the methodological quality of published systematic reviews of adverse events with surgical interventions. The potential predictors of the methodological quality were also analyzed.

## Methods

The current study is one of the series of a project that aimed to improve the evidence-based practice of systematic reviews of adverse events. A protocol of the project was developed in advance (supplementary file), which also involves some context referred to the current study [25]. This study was designed, conducted, and reported follows the Strengthening the Reporting of Observational Studies in Epidemiology (STROBE) Statement [26]; the following domains are not feasible for the current study: 1) Describe any sensitivity analyses (Reason: no need); 2) Indicate the number of participants with missing data for each variable of interest (Reason: no missing data). The details of the reporting are presented in supplementary files.

### Eligible studies

Systematic reviews and meta-analyses of surgical interventions with safety as the exclusive outcome were of interest. A surgical intervention is defined as a therapeutic or adjunctive invasive intervention performed by a trained clinician using hands, instruments, and/or devices. It includes operative, radiological, and endoscopic procedures [27, 28]. We considered any population that received surgical interventions with any types of safety outcomes. The comparison could be any other types of interventions (e.g., surgery, drug) or a placebo. However, we did not consider systematic reviews combined with original research because such studies generally put more focus on the original research. We took the definition of a systematic review by the Cochrane Collaboration, which refers to a type of review that attempts to identify, appraise, and synthesize all empirical evidence that meets pre-specified eligibility criteria to answer a specific research question [29]. However, in practice, some of the four key components in the definition may be missing, especially for non-Cochrane reviews. Therefore, we treated those with a pre-specified eligibility criterion, clear identification (literature search and screen) process, and synthesis scheme (qualitative or quantitative) as systematic reviews.

### Sample size estimation

To ensure the representativeness and sufficient statistical power, we estimated the minimum sample size (N) needed for the current study. This was done by setting 3 plausible proportions (*p* = 0.1, 0.3, 0.5) of adherence of the methodological items and a margin error (E) of 0.1 by the formula: *N* ***=*** 1.96^2^*p*(1 − *p*)/*E*^2^ [30]. As a result, the minimal sample size required by the current study ranged from 35 to 96 under different settings.

### Literature search and screening

We searched PubMed for eligible systematic reviews and meta-analyses from 1st-Jan, 2015 to 1st-Jan, 2020 without language restriction. This time frame was arbitrary, while we expected that the samples could be representative. In addition, systematic reviews in recent years were more time-sensitive for clinical practice. The literature search was done by a well-trained author. The search strategy was developed by a librarian based on the discussion with the team members, which was presented in the supplementary materials.

Two authors screened the records through the Rayyan application (https://rayyan.qcri.org/), which allowed the project manager to blind the two authors during the screening process. Titles and abstracts were first viewed to exclude studies that obviously did not meet the eligibility criteria. In this step, only those excluded by both of the screen authors were excluded. Then the full texts of the remaining studies were further reviewed for a final decision. Any disagreements were solved by discussing with the lead author. The agreement rate between the two authors during the full-text process was documented.

### Data extraction

The following baseline characteristics were extracted by two authors independently: name of first author, publication date, region of corresponding author, language of the review, any reporting and methodological guidelines referred in the review, number of included studies, type of included studies, type of the review (quantitative or qualitative), methods for rating evidence (e.g., Grading of Recommendations, Assessment, Development and Evaluations, GRADE [31]), and funding information. Then these data were double-checked, and any disagreements were solved by discussion.

### Assessment of methodological quality

We used the AMSTAR 2.0 instrument to assess the methodological quality of eligible systematic reviews and meta-analyses (see supplementary materials) [32]. It was primarily designed for evaluating systematic reviews of randomized or non-randomized studies of healthcare interventions [32]. Therefore, it is a valid tool to assess the methodological quality. The AMSTAR 2.0 instrument contains 16 domains with 7 assigned (item 2, 4, 7, 9, 11, 13, 15) as critical domains [32]. Any methodological weakness of the 7 domains would have a great impact on the quality of the systematic review.

The global rating of the methodological quality (high, moderate, low, critically low) of a systematic review was judged by the counts of identified critical and non-critical weaknesses. In brief, high quality requires no or only one non-critical weakness; moderate quality refers to two or more non-critical weaknesses but without critical weakness; low quality means one critical weakness with or without non-critical weaknesses; and those with two or more critical weaknesses were rated as critically low [32]. It should be noted that among the 16 domains, 3 were (11, 12, 15) not applicable for qualitative systematic reviews. The rating of the global methodological quality of qualitative systematic reviews was then based on the remaining 8 non-critical items and 5 critical items (item 2, 4, 7, 9, 13).

For each domain, there were two basic responses, i.e., “Yes” and “No”. Some of the domains may have an additional response as “Partially Yes”. A list of the required components was available to help raters qualitatively make a judgment for a certain domain. For example, item 4 (Did the review authors use a comprehensive literature search strategy?) had three responses: “Yes”, “Partial Yes”, and “No.” The “Partial Yes” option required three components: searching at least 2 databases, providing keyword and/or search strategy, justifying publication restrictions (e.g., language). For the “Yes” option, more components were needed, such as searching the reference lists, searching trial/study registries, etc. Similar to item 4, the boundary between “Partial Yes” and “Yes” was clear for most of the items. However, for domain 8 (Did the review authors describe the included studies in adequate detail?), there was no clear indicator to distinguish “Partially Yes” and “Yes”. The “Yes” option required that all the listed five components should be specified “in detail” [32]; the “Partially Yes” option also required the five components but did not require them “in detail”. There was no clear boundary for determining whether a component was “in detail” or not. We have contacted the lead author of AMSTAR 2.0 for clarification but failed to receive a response, so we rated all eligible systematic reviews that described the required components of this domain as “Partially Yes” to make a conservative evaluation [33]. This did not impact the rating of the methodological quality according to the rule of AMSTAR 2.0 [32].

The methodological quality was assessed by two well-trained authors through the Access software (Microsoft, USA), one methodologist and one anesthetist, and further checked by the lead author. Any disagreements were solved by discussion until consensus was achieved.

### Data analysis

Our primary interests were the number of weaknesses (i.e., those assigned as “No”) and the global methodological quality of each eligible systematic review. The median number as well as the interquartile range (IQR) were used to measure the central tendency and variability [30]. Both the counts of “No” from all domains and those from the critical domains were summarized.

Our secondary interest was the potential difference in the proportion of domain-specified weaknesses stratified by some pre-specified variables. These variables included: any reporting or methodology guidance referred in the review (yes vs. no), publication date (2018–2019 vs. 2015–2017), and development of protocol (yes vs. no). Considering that the development of protocol is already a part of AMSTAR 2.0, we removed the domain and only compared the remaining 15 domains under this variable. We also compared the proportion of weaknesses of systematic reviews with meta-analyses to those without meta-analyses for applicable domains. As aforementioned, for qualitative systematic reviews, three items (items 11, 12, and 15) were not applicable, which were not compared. For the publication date, considering the time lag, we used 2018 as the cut-off as the AMSTAR 2.0 was released in 2017 [32]. The proportion difference (PD) was used to measure the potential difference since this effect estimator has been proven to be valid to deal with potential zero-events in a single group or both groups [29]. The two-tailed z-test was used for the test of statistical significance. Since all the above variables can be obtained from the systematic reviews, we expect no missing data involved.

We used the MetaXL software (version 5.3, EpiGear, Australia) to run the analyses and the Excel 2013 (Microsoft, USA) to visualize the results. The level of statistical significance was pre-specified as alpha = 0.05(two-sides).

## Results

### Baseline characteristics

**Fig. 1:**
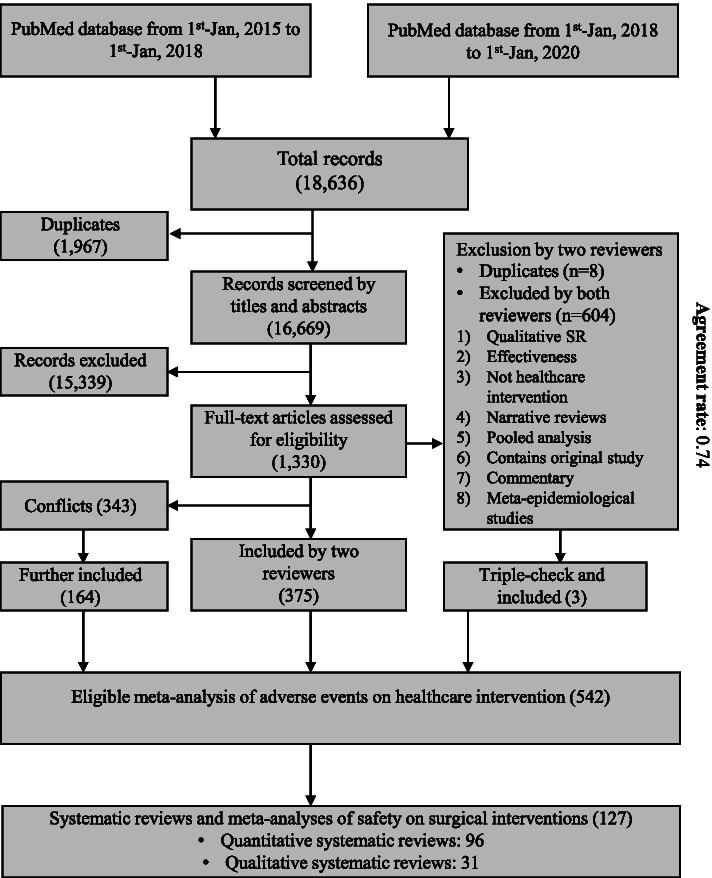
Flow plot of literature search and screening

Through the primary literature search, we obtained 18,636 records, where 1967 were identified as duplicates. For the 16,669 records that were viewed by titles and abstracts, 15,399 were excluded. From the remaining 1330 records, we identified 542 systematic reviews of interventions on safety outcomes, where 127 (19.48%) referred to surgical interventions (Fig. 1). The agreement rate was 0.74 in the full-text screening process. The details of excluded studies during the full-text screen process have been documented in our previous work [25]. According to the minimal requirement, the sample size of the current study could be sufficient.

Table 1 presents the baseline characteristics of these systematic reviews. Among the 127 eligible reviews, 96 (75.59%) were quantitative reviews (with meta-analyses), while 31 (24.41%) were qualitative reviews (without meta-analyses). Also, 81 (63.78%) were published between 2015 and 2017, and 46 (36.22%) were published between 2018 and 2019 (4 were published in issue in 2020 but online first in 2019). For the language, we identified 125 (98.43%) used English while 2 (1.57%) did not (1 Spanish and 1 French). There were 74 (58.27%) systematic reviews that referred to at least one guideline to design and conduct the study or report the results, while 41.73% failed to refer to any guideline. Only 5 (3.94%) reviews referred to a methodological guideline to design and conduct the study, and thus was not further analyzed.

**Table 1:** Basic characteristics of included systematic reviews on adverse events

Basic characteristics	No. of systematic reviews (***N*** = 127)
**Year**	
2015–2017	81 (63.78%)
2018–2019 (4 published in 2020 were online first in 2019)	46 (36.22%)
**Region of corresponding author**	
America (North and South)	32 (25.20%)
Asia	35 (27.56%)
European	54 (42.52%)
Oceania	6 (4.72%)
**Reporting guideline claimed by review authors**	
PRISMA	64 (50.39%)
MOOSE	5 (3.94%)
Cochrane Handbook^a^	3 (2.36%)
PRISMA and MOOSE	1 (0.79%)
PRISMA-P^a^	1 (0.79%)
No	53 (41.73%)
**Use of AMSTAR tool**	
Yes	5 (3.94%)
No	122 (96.06%)
**Language**	
English	125 (98.43%)
Other languages	2 (1.57%)
**Type of systematic reviews**	
Quantitative review (with meta-analysis)	96 (75.59%)
Narrative review (without meta-analysis)	31 (24.41%)
**Type of study for meta-analysis**	
Randomized controlled trial (RCT)	47 (37.01%)
Non-randomized study of intervention (NRSI)	25 (19.69%)
Both RCT and NRSI	46 (36.22%)
Not reported	9 (7.09%)
**Study number (Median, first and third quartiles)**	14 (8 to 28)
2–10	48 (37.80%)
11–20	36 (28.35%)
21–30	18 (14.17%)
> 30	25 (19.69)
**Funding**	
Non-profit	28 (22.05%)
Profit	2 (1.57%)
Not funded	58 (45.67%)
Not reported	39 (30.71%)
**Use of GRADE**	
Yes	13 (10.24%)
No	1144 (89.76%)

*GRADE* Grading of Recommendations, Assessment, Development and Evaluations [31]

^a^Please note that the Cochrane handbook is not a reporting guideline, and PRISMA-P is designed for the development of Protocol of systematic review not for systematic reviews. These two would not normally be appropriate guidelines for reporting

In addition, 47 (37.01%) systematic reviews included randomized controlled trials (RCTs) only, 25 (19.69%) included non-randomized studies of intervention (NRSI) only, and 46 (36.22%) included both types of studies. The median number of included studies was 14 (IQR: 8 to 28); 79 (62.2%) reviews included at least 10 studies. Funding information was available in 88 (69.29%) systematic reviews, where 28 (22.05%) received non-profit funding, 2 (1.57%) received profit funding, and the remaining 58 (45.67%) did not receive any funding support.

### Methodological quality and weakness

**Fig. 2:**
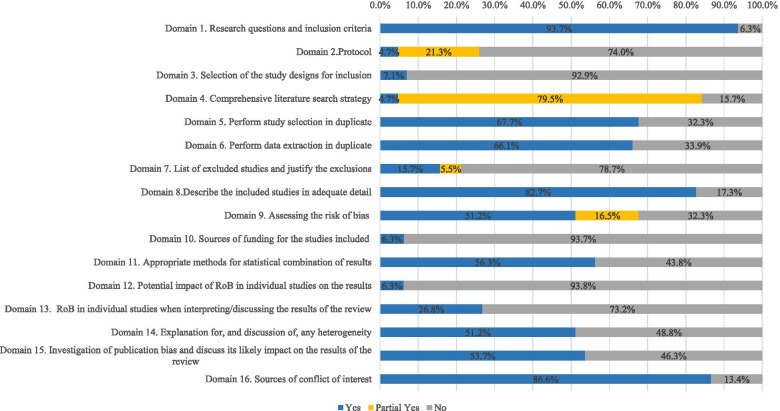
Methodological quality of 127 systematic reviews. Domains 11, 12, and 15 do not include the 31 qualitative systematic reviews without meta-analyses

Figure 2 presents the methodological quality of each domain of the 127 systematic reviews. For the 16 domains, only 4 (1, 4, 8, and 16) in most (> 80%) of the systematic reviews showed no methodological weakness. Of the four domains, 1 (domain 4) was a critical domain. In addition, 6 domains with about a half to two-thirds of the systematic reviews showed no methodological weakness; they were domains 5, 6, 9, 11, 14, and 15, where 3 (9, 11, and 15) were critical ones. For the remaining 6 domains (2, 3, 7, 10, 12, 13), most (70%) of the systematic reviews had methodological weaknesses. Again, 3 (2, 7, 13) were critical domains.

**Fig. 3:**
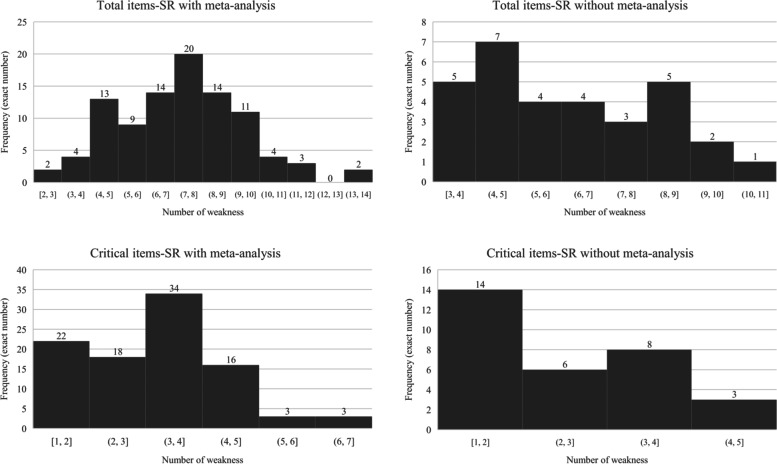
Distribution of methodological weaknesses. SR: systematic review

In terms of global methodological quality, none of the 127 systematic reviews could be rated as “high”. In contrast, they were either rated as “low” (*n* = 18, 14.17%) or as “critically low” (*n* = 109, 85.83%). The median number of methodological weaknesses of these systematic reviews was 8 (IQR: 6 to 9), of which 4 (IQR: 2 to 4) were critical weaknesses. This meant that half of the systematic reviews had 8 or more methodological weaknesses in total, and 4 or more for critical ones. Similarly, 75% of the systematic reviews had 6 or more methodological weaknesses (Fig. 3).

### Factors related to methodological weaknesses

**Fig. 4:**
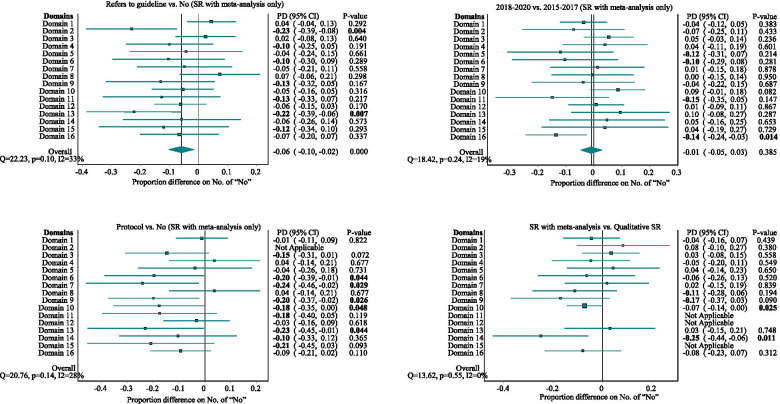
Factors that may relate to the methodological weaknesses. Notes: The domains with absolute PDs larger than 0.1 (in absolute magnitude) and/or with *p*-values less than 0.05 were highlighted in bold. The variable development of protocol is exactly the domain 2 in AMSTAR 2.0 instrument. Therefore, we did not compare the rate of “No” for item 2 by this variable. Similarly, domains 11, 12, and 15 were not suitable for qualitative systematic reviews without meta-analysis

Figure 4 shows the comparisons of the pre-defined factors on the proportion of methodological weaknesses. In general, the systematic reviews that referred to any reporting guidelines (e.g., domain 13, PD = -0.22, 95% CI: − 0.39, − 0.06; *p* = 0.01), developed a protocol in advance (e.g., domain 6, PD = -0.20, 95% CI: − 0.39, − 0.01; *p* = 0.04), and applied a quantitative analysis (e.g., domain 14, PD = -0.25, 95% CI: − 0.44, − 0.06, *p* = 0.01) tended to have less methodological weaknesses in some domains. However, for some domains, there were no obvious improvement (e.g., domain 8, PD = 0.04, 95%CI: − 0.14, 0.21; *p* = 0.68; with protocol vs. without). In addition, more recent systematic reviews showed slightly fewer methodological weaknesses, but in most of the domains, the difference was not significant from both clinical and statistical perspectives.

## Discussion

In this study, we conducted a comprehensive meta-epidemiological survey on the methodological quality of systematic reviews on adverse events of surgical interventions. We found that the methodological quality of these systematic reviews was poor, and none could be rated as “high” in terms of the quality rating. These reviews were either rated as “low” or as “critically low”. Our study suggested that half of these systematic reviews had at least 8 methodological weaknesses, 75% had at least 6 methodological weaknesses, and many of these weaknesses were critical.

This study also found that systematic reviews that used any reporting guideline and (or) developed a protocol in advance were less likely to have methodological weaknesses in most of the domains (13 out of 16), although some of which were not statistically significant. These findings concurred with previous studies [24, 34–36]. These findings reinforced the importance of protocol and reporting guideline, and thus future systematic review authors are highly recommended to develop a protocol in advance and report their research by referring to a relevant guideline (e.g., the PRISMA 2020 [37]).

We noticed that only 56.3% (*n* = 54) systematic reviews (with meta-analyses) employed appropriate synthesis methods for the meta-analyses and 43.8% (*n* = 42) did not. This was mainly because more than half (*n* = 27, 64.29%) of these unpleasant systematic reviews did not consider the potential evidence from studies with no events. Such studies are important sources of evidence; our previous studies have shown that discarding such studies could lead to about 10% of the meta-analyses with altered conclusions [38]. Moreover, 12 (28.57%) of these unpleasant reviews combined the results of RCTs and NRSIs together, which has been criticized [30, 32, 33]. We also recorded inappropriate synthesis methods such as failure to address substantial heterogeneity and employing a wrong synthesis method (i.e., unweighted average). These serious issues should be particularly examined in future systematic reviews because an inappropriate synthesis of the evidence could lead to dramatically wrong results with misleading conclusions.

In this survey, we collected the systematic reviews of adverse events of surgical interventions in the past 5 years. To the best of our knowledge, this is the first meta-epidemiology survey focusing on the methodological quality of such systematic reviews. The rigorous implementation of our study ensured that the findings were credible. The findings will provide useful information and evidence foundation for clinical practice guideline developers and policy makers to better form the recommendations. The findings will also give a wake-up call for systematic review authors to avoid some frequently neglected methodological issues.

Several limitations should be highlighted. We used the AMSTAR 2.0 instrument to assess the methodological quality of the systematic reviews. It is hard to cover all important methodological domains, and there may be some other unmeasured methodological issues (e.g., data extraction error [39]). Therefore, the current study may overestimate the methodological quality of the systematic reviews. Another major limitation is that the assessment of the methodological quality was entirely based on how the related information was originally reported in the systematic reviews. Some authors possibly did not report the information that they consider to be unimportant. In this situation, the methodological quality of the systematic reviews may be underestimated. Third, we only collected systematic reviews and meta-analyses of the recent 5 years and failed to include those published much earlier. The findings of the current study may not well represent those published much earlier. These limitations were inevitable and could potentially bring some biases to our results.

## Conclusions

Based on the findings of our study, the methodological quality of systematic reviews of adverse events with surgical interventions was generally poor. Half of them had 8 or more methodological weaknesses, three-fourths of them had 6 or more methodological weaknesses, and many of the weaknesses were critical weaknesses. Some easy-to-implement measures could be used to improve the methodological quality, such as referring to any reporting guidance and developing a detailed research protocol in advance. Further efforts are needed to enhance the methodological quality of these systematic reviews.

## Supplementary Information

**Additional file 1. **STROBE Statement—Checklist of items that should be included in reports of *cross-sectional studies.*

**Additional file 2.** PROTOCOL.

**Additional file 3.** Original data.

## Data Availability

All data generated or analysed during this study are included in this published article and its supplementary information files.
