# Supplementary material for: Methodological quality for systematic reviews of adverse events with surgical interventions: a cross-sectional survey
Source: BMC Med Res Methodol. 2021 Oct 25;21:223. doi: 10.1186/s12874-021-01423-6 (PMC8543966; doi:10.1186/s12874-021-01423-6)
Supplement: Supplementary file 2 — Additional file 2. PROTOCOL. [file 12874_2021_1423_MOESM2_ESM.docx]

**Title: PROTOCOL**

**Handling methods for zero-events studies in meta-analyses of adverse events: meta-epidemiological study**

**Drafted by:** Chang Xu^1,2^

1. Department of Population Medicine, College of Medicine, Qatar University, Al Jamiaa Street, P. O. Box 2713, Doha, Qatar;
2. Chinese Evidence-based Medicine Center, West China Hospital, Sichuan University, Chengdu, China;

## ^🖂^Correspondence to:

Dr. Chang Xu - [xuchang2016@runbox.com](mailto:xuchang2016@runbox.com)

Department of Population Medicine, College of Medicine, Qatar University, Al Jamiaa Street, P. O. Box 2713, Doha, Qatar;

## Research question

In the era of evidence-based medicine, systematic review and meta-analysis contributes the most important source of evidence and has been widely used to assess the effectiveness and safety of healthcare intervention. In a meta-analysis, data from available studies on the same topic are quantitively synthesized as an effort to reduce the uncertainty and increase the credibility. It has been well-recognized that meta-analysis could achieve reasonable statistical properties for efficacy assessment; however, for safety assessment, due to the potential low events rate and limited sample size, the observed events tend to be rare and often zero, make the data synthesis challengeable.

For studies with zero-events in a single arm, several well-established methods (e.g. Peto Odds ratio, continuity correction, Mantel–Haenszel) were available to synthesis the information of such studies into meta-analysis, and there is a unanimous agreement that such studies should be incorporated into meta-analysis. For studies with zero-events in both arms, researchers either discard them from the meta-analysis or incorporate them through some sophisticated methods (e.g. Bayesian, generalized linear mixed model (GLMM)). Due to the nature of such data, the results were most likely unstable and susceptive to the methods employed, which further largely impacted the credibility of the conclusions of a meta-analysis. Understanding how such studies were handled is necessarily important, which will have implications for further guidelines, practice and policy.

## Searches

We will search PubMed for all related meta-analyses of randomized controlled trials for adverse events. The primary search strategy was developed by a librarian, and then discussed with the lead author for further adjustment (see Supplements). Considering the huge number of meta-analysis published in recent decades, we will limit our search to recent 5 years (2015/01/01 to 2020/01/01). To better understand how studies of zero-events were handled in meta-analyses of adverse events in the past, we will also include our previous dataset for meta-analyses of rare events (January 2008 to April 2011).

Grey literature will not be considered as we only aimed at published meta-analyses. We hypothesized that these published meta-analyses would be of more deliberate in dealing with zero-events studies after strict peer review process. Considering the sample would be representative, we will not use hand search for the reference lists of each systematic reviews and meta-analyses.

## Types of study to be included

Meta-analyses of randomized controlled trials with adverse events as the unique outcome(s). We defined adverse events as “any unintended effect or effects of an intervention”, which could be an adverse event, adverse effect, adverse reaction, harms, or complications associated with any healthcare intervention. And all “adverse events” should be binary outcomes. We will not consider those meta-analyses assessing both effectiveness and safety; we will also not consider meta-analyses of incidence or prevalence of adverse events that with only a single arm. Systematic reviews without a meta-analysis will be excluded.

## Context

This study will focus on how zero-events studies were dealt with in meta-analyses of adverse events. We will summarize this information for zero-events in a single arm and zero-events in both arms separately, with a special focus on the later. These include:

***Main information: Meta-analysis with zero-events in both arms***

1. Whether studies with zero-events in both arms were synthesized;
2. Which effect estimator (e.g. OR, RR, RD) was used;
3. Which synthesis method was used;
4. Whether a sensitivity analysis was employed through at least one different synthesis methods;
5. For those failed to synthesize studies with zero-events in both arms, whether any further analysis was conducted to evaluate the potential impact (of excluding such studies) on the results;

***Main information: Meta-analysis with zero-events in*** ***single arm***

1. which effect estimator (e.g. OR, RR, RD) was used;
2. which synthesis method was used;
3. whether a sensitivity analysis was employed through at least one different synthesis methods;

***Other information***

1. How between-study heterogeneity was measured, and if conducted, whether a substantial heterogeneity was detected (According to Cochrane’s standard, for example, I^2^ > 50%);
2. How publication bias was measured, and if conducted, whether a publication bias was detected (According to the measuring method itself, for p-value driven method, we set the cut-off point as p = 0.10);

We will further categorize the methods used in meta-analyses as frequentist and Bayesian framework, where for each framework, the detailed methods will be summarized. Such information will be compared for meta-analyses published in recent 5 years and those published in 2008 to 2011.

## Main outcome(s)

The primary outcome is the percentage (and ranks) of the methods used for dealing with zero-events studies. The co-primary outcome is the proportion of meta-analyses that zero-events studies were correctly dealt with over years. We defined those meta-analyses discard zero-events studies as incorrectly dealt with.

## Assessment of risk of bias

The PRISMA-harms and AMSTAR 2.0 checklists will be used to assess the potential bias (quality) on reporting and methodology. This will be treated as separate paper(s).

## Data extraction (selection and coding)

Two authors will screen the literature for eligibility independently through the Rayyan online app, since this app allows a restrict blinding for the two rators to ensure the process was independent. The titles and abstracts will be first screened and those obviously not meet the criteria will be excluded; then the full-text will be checked for final decision.

Data exaction (above mentioned information in “Context” section) will be employed by an experienced statistician (master degree), and will be double checked by the lead author. Baseline characteristics such as author name, number of authors, publication year, region of first author, use of reporting guidance, use of GRADE, funding information, type of meta-analyses (e.g. generic, network) will also be extracted.

## Analysis of subgroups or subsets

- Eligible meta-analyses published in recent 5 years (2015/01/01 to 2020/01/01)
- Eligible meta-analyses published from Jan-2008 to April-2011.

## Contact details for further information

[xuchang2016@runbox.com](mailto:xuchang2016@runbox.com)

## Conflicts of interest

We declare no conflict of interest.

## Funding

This study did not receive any financial supporting.

Stage of review
Review Ongoing: Literature search has been conducted and is currently on literature screen (By 28^th^-July, 2020).

**Deviations of protocol and the article**

Some minor changes were made as follows: First, in the protocol, we planned to collect the information of how between-study heterogeneity was measured and how publication bias was detected. This was not done as such information was already represented in our other recent study. Second, one of our aims was to categorize the methods for dealing with zero-events studies in terms of the frequentist and Bayesian frameworks. However, we only identified one study using Bayesian methods, so we removed this aim. Third, our primary “population” was meta-analyses of randomized controlled trials (RCTs) on intervention safety. However, during the search procedure, we realized that many meta-analyses with non-randomized studies of intervention (NRSI) also faced the zero-events problem. We therefore decided to include meta-analyses with non-randomized studies as well.

## Title: Supplements-Search strategy (PubMed, conducted at 28^th^-July, 2020)

1. "Systematic Reviews as Topic"[Mesh] OR "Systematic Review" [Publication Type] OR "Meta-Analysis as Topic"[Mesh] OR "Meta-Analysis" [Publication Type] OR "meta-analysis"[Title/Abstract] OR "meta analysis"[Title/Abstract] OR "systematic review"[Title/Abstract]
2. "randomized controlled trials as topic"[MeSH Major Topic] OR "clinical trials as topic"[MeSH Major Topic] OR "controlled clinical trials as topic"[MeSH Major Topic]
3. "randomized controlled trial*"[Title/Abstract] OR "controlled clinical trial*"[Title/Abstract] OR "clinical trial*"[Title/Abstract] OR "controlled trial*"[Title/Abstract] OR "trial*"[Title/Abstract]
4. "safety"[Title/Abstract] OR "harm*"[Title/Abstract] OR safe[Title/Abstract] OR poisoning[Title/Abstract] OR toxicity[Title/Abstract] OR tolerability[Title/Abstract] OR "complication*"[Title/Abstract] OR "adverse event*"[Title/Abstract] OR "adverse outcome*"[Title/Abstract] OR "untoward effect*"[Title/Abstract] OR "side effect*"[Title/Abstract] OR adverse n2 reaction[Title/Abstract]
5. #2 or #3
6. #1 AND #4 AND #5
7. Protocol[Title] OR overview [Title] OR "narrative review" [Title]
8. #6 NOT #7
9. (#8) AND (("2018/01/02"[Date - Publication]: "2020/01/01"[Date - Publication])) AND (humans[Filter]) Filters: Humans
10. (#8) AND (("2015/01/01"[Date - Publication]: "2018/01/01"[Date - Publication])) AND (humans[Filter]) Filters: Humans
11. #9 or # 10

## List of included studies

**96 systematic reviews with meta-analyses**

1. Abdelaziz HK, Megaly M, Debski M, Rahbi H, Kamal D, Saad M, Wiper A, More R, Roberts DH. Meta-Analysis Comparing Percutaneous to Surgical Access in Trans-Femoral Transcatheter Aortic Valve Implantation. Am J Cardiol. 2020 Apr 15;125(8):1239-1248.
2. Abdelaziz HK, Megaly M, Debski M, Rahbi H, Kamal D, Saad M, Wiper A, More R, Roberts DH. Meta-Analysis Comparing Percutaneous to Surgical Access in Trans-Femoral Transcatheter Aortic Valve Implantation. Am J Cardiol. 2020 Apr 15;125(8):1239-1248.
3. Ahmed AM, Moahammed AT, Mattar OM, Mohamed EM, Faraag EA, AlSafadi AM, Hirayama K, Huy NT. Surgical treatment of diverticulitis and its complications: A systematic review and meta-analysis of randomized control trials. Surgeon. 2018 Dec;16(6):372-383.
4. Almpani K, Papageorgiou SN, Papadopoulos MA. Autotransplantation of teeth in humans: a systematic review and meta-analysis. Clin Oral Investig. 2015 Jul;19(6):1157-79.
5. Ando T, Ashraf S, Villablanca PA, Telila TA, Takagi H, Grines CL, Afonso L, Briasoulis A. Meta-Analysis Comparing the Incidence of Infective Endocarditis Following Transcatheter Aortic Valve Implantation Versus Surgical Aortic Valve Replacement. Am J Cardiol. 2019 Mar 1;123(5):827-832.
6. Araujo Júnior E, Eggink AJ, van den Dobbelsteen J, Martins WP, Oepkes D. Procedure-related complications of open vs endoscopic fetal surgery for treatment of spina bifida in an era of intrauterine myelomeningocele repair: systematic review and meta-analysis. Ultrasound Obstet Gynecol. 2016 Aug;48(2):151-60.
7. Balk EM, Earley A, Avendano EA, Raman G. Long-Term Health Outcomes in Women With Silicone Gel Breast Implants: A Systematic Review. Ann Intern Med. 2016 Feb 2;164(3):164-75.
8. Barzilay E, Gadot Y, Koren G. Safety of vaginal delivery in very low birthweight vertex singletons: a meta-analysis. J Matern Fetal Neonatal Med. 2016 Nov;29(22):3724-9.
9. Beger HG, Siech M, Poch B, Mayer B, Schoenberg MH. Limited surgery for benign tumours of the pancreas: a systematic review. World J Surg. 2015 Jun;39(6):1557-66.
10. Berstock JR, Blom AW, Beswick AD. A systematic review and meta-analysis of complications following the posterior and lateral surgical approaches to total hip arthroplasty. Ann R Coll Surg Engl. 2015 Jan;97(1):11-6.
11. Biardeau X, Zanaty M, Aoun F, Benbouzid S, Peyronnet B. Voies d'abord et complications des bandelettes sous-urétrales synthétiques chez la femme : revue systématique de la littérature et méta-analyse [Approach and complications associated with suburethral synthetic slings in women: Systematic review and meta-analysis]. Prog Urol. 2016 Mar;26(4):254-69.
12. Bouza C, López-Cuadrado T, Almendro N, Amate JM. Safety of balloon kyphoplasty in the treatment of osteoporotic vertebral compression fractures in Europe: a meta-analysis of randomized controlled trials. Eur Spine J. 2015 Apr;24(4):715-23.
13. Brener MI, Bush A, Miller JM, Hasan RK. Influence of radial versus femoral access site on coronary angiography and intervention outcomes: A systematic review and meta-analysis. Catheter Cardiovasc Interv. 2017 Dec 1;90(7):1093-1104.
14. Brotis AG, Tasiou A, Paterakis K, Tzerefos C, Fountas KN. Complications Associated with Surgery for Thoracic Disc Herniation: A Systematic Review and Network Meta-Analysis. World Neurosurg. 2019 Dec;132:334-342.
15. Bundhun PK, Janoo G, Chen MH. Bleeding events associated with fibrinolytic therapy and primary percutaneous coronary intervention in patients with STEMI: A systematic review and meta-analysis of randomized controlled trials. Medicine (Baltimore). 2016 Jun;95(23):e3877.
16. Bundhun PK, Bhurtu A, Pursun M, Soogund MZS, Teeluck AR, Huang WQ. Long-term (2-5 years) adverse clinical outcomes associated with ZES versus SES, PES and EES: A Meta-Analysis. Sci Rep. 2017 Jul 25;7(1):6385.
17. Carloni R, Naudet F, Chaput B, de Runz A, Herlin C, Girard P, Watier E, Bertheuil N. Are There Factors Predictive of Postoperative Complications in Circumferential Contouring of the Lower Trunk? A Meta-Analysis. Aesthet Surg J. 2016 Nov;36(10):1143-1154.
18. Ceresoli M, Tamini N, Gianotti L, Braga M, Nespoli L. Are endoscopic loop ties safe even in complicated acute appendicitis? A systematic review and meta-analysis. Int J Surg. 2019 Aug;68:40-47.
19. Chitragari G, Schlosser FJ, Ochoa Chaar CI, Sumpio BE. Consequences of hypogastric artery ligation, embolization, or coverage. J Vasc Surg. 2015 Nov;62(5):1340-7.e1.
20. Crawley B, Dehom S, Tamares S, Marghalani A, Ongkasuwan J, Reder L, Ivey C, Amin M, Fritz M, Pitman M, Tulunay-Ugur O, Weissbrod P. Adverse Events after Rigid and Flexible Endoscopic Repair of Zenker's Diverticula: A Systematic Review and Meta-analysis. Otolaryngol Head Neck Surg. 2019 Sep;161(3):388-400.
21. Cusimano MC, Simpson AN, Dossa F, Liani V, Kaur Y, Acuna SA, Robertson D, Satkunaratnam A, Bernardini MQ, Ferguson SE, Baxter NN. Laparoscopic and robotic hysterectomy in endometrial cancer patients with obesity: a systematic review and meta-analysis of conversions and complications. Am J Obstet Gynecol. 2019 Nov;221(5):410-428.e19.
22. Dall'Asta A, Ghi T, Pedrazzi G, Frusca T. Does vacuum delivery carry a higher risk of shoulder dystocia? Review and meta-analysis of the literature. Eur J Obstet Gynecol Reprod Biol. 2016 Sep;204:62-8.
23. de Carvalho ALR, Vital RB, de Lira CCS, Magro IB, Sato PTS, Lima LHN, Braz LG, Módolo NSP. Laryngeal Mask Airway Versus Other Airway Devices for Anesthesia in Children With an Upper Respiratory Tract Infection: A Systematic Review and Meta-analysis of Respiratory Complications. Anesth Analg. 2018 Oct;127(4):941-950.
24. de Resende JA Júnior, Cavalini LT, Crispi CP, de Freitas Fonseca M. Risk of urinary retention after nerve-sparing surgery for deep infiltrating endometriosis: A systematic review and meta-analysis. Neurourol Urodyn. 2017 Jan;36(1):57-61.
25. Diederen M, Gommers J, Wilkinson C, Turnbull D, Mol B. Safety of the balloon catheter for cervical ripening in outpatient care: complications during the period from insertion to expulsion of a balloon catheter in the process of labour induction: a systematic review. BJOG. 2018 Aug;125(9):1086-1095.
26. Elgendy IY, Mahmoud AN, Brilakis ES, Bavry AA. Drug-eluting stents versus bare metal stents for saphenous vein graft revascularisation: a meta-analysis of randomised trials. EuroIntervention. 2018 Jun 20;14(2):215-223.
27. Geminiani A, Tsigarida A, Chochlidakis K, Papaspyridakos PV, Feng C, Ercoli C. A meta-analysis of complications during sinus augmentation procedure. Quintessence Int. 2017;48(3):231-240.
28. Geng HZ, Nasier D, Liu B, Gao H, Xu YK. Meta-analysis of elective surgical complications related to defunctioning loop ileostomy compared with loop colostomy after low anterior resection for rectal carcinoma. Ann R Coll Surg Engl. 2015 Oct;97(7):494-501.
29. Ghayoumi P, Kandemir U, Morshed S. Evidence based update: open versus closed reduction. Injury. 2015 Mar;46(3):467-73.
30. Ghumman SS, Weinerman J, Khan A, Cheema MS, Garcia M, Levin D, Suri R, Prasad A. Contrast induced-acute kidney injury following peripheral angiography with carbon dioxide versus iodinated contrast media: A meta-analysis and systematic review of current literature. Catheter Cardiovasc Interv. 2017 Sep 1;90(3):437-448.
31. Giacoppo D, Colleran R, Cassese S, Frangieh AH, Wiebe J, Joner M, Schunkert H, Kastrati A, Byrne RA. Percutaneous Coronary Intervention vs Coronary Artery Bypass Grafting in Patients With Left Main Coronary Artery Stenosis: A Systematic Review and Meta-analysis. JAMA Cardiol. 2017 Oct 1;2(10):1079-1088.
32. Gong W, Li A, Ai H, Shi H, Wang X, Nie S. Safety of early discharge after primary angioplasty in low-risk patients with ST-segment elevation myocardial infarction: A meta-analysis of randomised controlled trials. Eur J Prev Cardiol. 2018 May;25(8):807-815.
33. Healy DA, Kimura S, Power D, Elhaj A, Abdeldaim Y, Cross KS, McGreal GT, Burke PE, Moloney T, Manning BJ, Kavanagh EG. A Systematic Review and Meta-analysis of Thrombotic Events Following Endovenous Thermal Ablation of the Great Saphenous Vein. Eur J Vasc Endovasc Surg. 2018 Sep;56(3):410-424.
34. Henriksen NA, Deerenberg EB, Venclauskas L, Fortelny RH, Garcia-Alamino JM, Miserez M, Muysoms FE. Triclosan-coated sutures and surgical site infection in abdominal surgery: the TRISTAN review, meta-analysis and trial sequential analysis. Hernia. 2017 Dec;21(6):833-841.
35. Hua J, He Z, Qian D, Meng H, Zhou B, Song Z. Duct-to-Mucosa Versus Invagination Pancreaticojejunostomy Following Pancreaticoduodenectomy: a Systematic Review and Meta-Analysis. J Gastrointest Surg. 2015 Oct;19(10):1900-9.
36. Inokuchi M, Otsuki S, Fujimori Y, Sato Y, Nakagawa M, Kojima K. Systematic review of anastomotic complications of esophagojejunostomy after laparoscopic total gastrectomy. World J Gastroenterol. 2015 Aug 28;21(32):9656-65.
37. Inokuchi M, Sugita H, Otsuki S, Sato Y, Nakagawa M, Kojima K. Laparoscopic distal gastrectomy reduced surgical site infection as compared with open distal gastrectomy for gastric cancer in a meta-analysis of both randomized controlled and case-controlled studies. Int J Surg. 2015 Mar;15:61-7.
38. Jiang J, Zou J, Ma H, Jiao Y, Yang H, Zhang X, Miao Y. Network Meta-analysis of Randomized Trials on the Safety of Vascular Closure Devices for Femoral Arterial Puncture Site Haemostasis. Sci Rep. 2015 Sep 8;5:13761.
39. Johnstone MS. Vertical Rectus Abdominis Myocutaneous Versus Alternative Flaps for Perineal Repair After Abdominoperineal Excision of the Rectum in the Era of Laparoscopic Surgery. Ann Plast Surg. 2017 Jul;79(1):101-106.
40. Katsanos K, Spiliopoulos S, Kitrou P, Krokidis M, Karnabatidis D. Risk of Death Following Application of Paclitaxel-Coated Balloons and Stents in the Femoropopliteal Artery of the Leg: A Systematic Review and Meta-Analysis of Randomized Controlled Trials. J Am Heart Assoc. 2018 Dec 18;7(24):e011245.
41. Klotz R, Probst P, Deininger M, Klaiber U, Grummich K, Diener MK, Weigand MA, Büchler MW, Knebel P. Percutaneous versus surgical strategy for tracheostomy: a systematic review and meta-analysis of perioperative and postoperative complications. Langenbecks Arch Surg. 2018 Mar;403(2):137-149.
42. la Chapelle CF, Swank HA, Wessels ME, Mol BW, Rubinstein SM, Jansen FW. Trocar types in laparoscopy. Cochrane Database Syst Rev. 2015 Dec 16;(12):CD009814.
43. Lalu MM, Fayad A, Ahmed O, Bryson GL, Fergusson DA, Barron CC, Sullivan P, Thompson C; Canadian Perioperative Anesthesia Clinical Trials Group. Ultrasound-Guided Subclavian Vein Catheterization: A Systematic Review and Meta-Analysis. Crit Care Med. 2015 Jul;43(7):1498-507.
44. Lewis SR, Butler AR, Parker J, Cook TM, Smith AF. Videolaryngoscopy versus direct laryngoscopy for adult patients requiring tracheal intubation. Cochrane Database Syst Rev. 2016 Nov 15;11(11):CD011136.
45. Li DF, Yang MF, Chang X, Wang NN, Tan FF, Xie HN, Fang X, Wang SL, Fan W, Wang JY, Yu ZC, Wei C, Xiong F, Liu TT, Luo MH, Wang LS, Li ZS, Yao J, Bai Y. Endocut Versus Conventional Blended Electrosurgical Current for Endoscopic Biliary Sphincterotomy: A Meta-Analysis of Complications. Dig Dis Sci. 2019 Aug;64(8):2088-2094.
46. Li L, Zhang Z, Yao Z, Wang H, Wang H, An H, Yao J. The impact of laryngeal mask versus other airways on perioperative respiratory adverse events in children: A systematic review and meta-analysis of randomized controlled trials. Int J Surg. 2019 Apr;64:40-48.
47. Lim G, Lin GH, Monje A, Chan HL, Wang HL. Wound Healing Complications Following Guided Bone Regeneration for Ridge Augmentation: A Systematic Review and Meta-Analysis. Int J Oral Maxillofac Implants. 2018 January/February;33(1):41–50.
48. Liu P, Lin H, Chen Y, Wu YS, Tang M, Liu C. Comparison of Metal and Plastic Stents for Preoperative Biliary Drainage in Resectable and Borderline Resectable Periampullary Cancer: A Meta-Analysis and System Review. J Laparoendosc Adv Surg Tech A. 2018 Sep;28(9):1074-1082.
49. Liu YX, Zhang Y, Huang JF, Wang L. Meta-analysis comparing the safety of laparoscopic and open surgical approaches for suspected adnexal mass during the second trimester. Int J Gynaecol Obstet. 2017 Mar;136(3):272-279.
50. Lv Z, Li Y, Wu Y, Qu Y. Surgical complications of primary rhegmatogenous retinal detachment: a meta-analysis. PLoS One. 2015 Mar 3;10(3):e0116493.
51. Maniotis C, Andreou C, Karalis I, Koutouzi G, Agelaki M, Koutouzis M. A systematic review on the safety of Prostar XL versus ProGlide after TAVR and EVAR. Cardiovasc Revasc Med. 2017 Mar;18(2):145-150.
52. Migliore M, Arezzo A, Arolfo S, Passera R, Morino M. Safety of single-incision robotic cholecystectomy for benign gallbladder disease: a systematic review. Surg Endosc. 2018 Dec;32(12):4716-4727.
53. Mohananey D, Sengodan P, Banerjee K, Kumar A, Jobanputra Y, Sankaramangalam K, Krishnaswamy A, Mick S, White JM, Svensson LG, Kapadia SR. Comparative analysis of cerebrovascular events in transcatheter and surgical aortic valve replacement: a systematic review and meta-analysis of randomised trials. EuroIntervention. 2018 May 20;14(1):69-77.
54. Morris BJ, Krieger JN. Does Circumcision Increase Meatal Stenosis Risk?-A Systematic Review and Meta-analysis. Urology. 2017 Dec;110:16-26.
55. Munster AB, Franchini AJ, Qureshi MI, Thapar A, Davies AH. Temporal trends in safety of carotid endarterectomy in asymptomatic patients: systematic review. Neurology. 2015 Jul 28;85(4):365-72.
56. Neto AS, Hemmes SN, Barbas CS, Beiderlinden M, Fernandez-Bustamante A, Futier E, Gajic O, El-Tahan MR, Ghamdi AA, Günay E, Jaber S, Kokulu S, Kozian A, Licker M, Lin WQ, Maslow AD, Memtsoudis SG, Reis Miranda D, Moine P, Ng T, Paparella D, Ranieri VM, Scavonetto F, Schilling T, Selmo G, Severgnini P, Sprung J, Sundar S, Talmor D, Treschan T, Unzueta C, Weingarten TN, Wolthuis EK, Wrigge H, Amato MB, Costa EL, de Abreu MG, Pelosi P, Schultz MJ; PROVE Network Investigators. Association between driving pressure and development of postoperative pulmonary complications in patients undergoing mechanical ventilation for general anaesthesia: a meta-analysis of individual patient data. Lancet Respir Med. 2016 Apr;4(4):272-80.
57. Osland E, Yunus RM, Khan S, Memon B, Memon MA. Late Postoperative Complications in Laparoscopic Sleeve Gastrectomy (LVSG) Versus Laparoscopic Roux-en-y Gastric Bypass (LRYGB): Meta-analysis and Systematic Review. Surg Laparosc Endosc Percutan Tech. 2016 Jun;26(3):193-201.
58. Pardal-Refoyo JL, Ochoa-Sangrador C. Bilateral recurrent laryngeal nerve injury in total thyroidectomy with or without intraoperative neuromonitoring. Systematic review and meta-analysis. Acta Otorrinolaringol Esp. 2016 Mar-Apr;67(2):66-74.
59. Parker M, Raval P, Gjertsen JE. Nail or plate fixation for A3 trochanteric hip fractures: A systematic review of randomised controlled trials. Injury. 2018 Jul;49(7):1319-1323.
60. Pecorelli N, Greco M, Amodeo S, Braga M. Small bowel obstruction and incisional hernia after laparoscopic and open colorectal surgery: a meta-analysis of comparative trials. Surg Endosc. 2017 Jan;31(1):85-99
61. Peng C, Ling Y, Ma C, Ma X, Fan W, Niu W, Niu J. Safety Outcomes of NOTES Cholecystectomy Versus Laparoscopic Cholecystectomy: A Systematic Review and Meta-Analysis. Surg Laparosc Endosc Percutan Tech. 2016 Oct;26(5):347-353.
62. Pergialiotis V, Mudiaga Z, Perrea DN, Doumouchtsis SK. De novo overactive bladder following midurethral sling procedures: a systematic review of the literature and meta-analysis. Int Urogynecol J. 2017 Nov;28(11):1631-1638.
63. Sandberg F, Viktorsdóttir MB, Salö M, Stenström P, Arnbjörnsson E. Comparison of major complications in children after laparoscopy-assisted gastrostomy and percutaneous endoscopic gastrostomy placement: a meta-analysis. Pediatr Surg Int. 2018 Dec;34(12):1321-1327.
64. Sathya C, Wayne C, Gotsch A, Vincent J, Sullivan KJ, Nasr A. Laparoscopic versus open pyloromyotomy in infants: a systematic review and meta-analysis. Pediatr Surg Int. 2017 Mar;33(3):325-333.
65. Schiphorst AH, Verweij NM, Pronk A, Borel Rinkes IH, Hamaker ME. Non-surgical complications after laparoscopic and open surgery for colorectal cancer - A systematic review of randomised controlled trials. Eur J Surg Oncol. 2015 Sep;41(9):1118-27.
66. Schulman AR, Popov V, Thompson CC. Randomized sham-controlled trials in endoscopy: a systematic review and meta-analysis of adverse events. Gastrointest Endosc. 2017 Dec;86(6):972-985.e3.
67. Shah K, Chaker Z, Busu T, Shah R, Osman M, Alqahtani F, Alkhouli M. Meta-Analysis Comparing Renal Outcomes after Transcatheter versus Surgical Aortic Valve Replacement. J Interv Cardiol. 2019 Apr 24;2019:3537256.
68. Shriver MF, Xie JJ, Tye EY, Rosenbaum BP, Kshettry VR, Benzel EC, Mroz TE. Lumbar microdiscectomy complication rates: a systematic review and meta-analysis. Neurosurg Focus. 2015 Oct;39(4):E6.
69. Sirker A, Kwok CS, Kotronias R, Bagur R, Bertrand O, Butler R, Berry C, Nolan J, Oldroyd K, Mamas MA. Influence of access site choice for cardiac catheterization on risk of adverse neurological events: A systematic review and meta-analysis. Am Heart J. 2016 Nov;181:107-119.
70. Sonalkar S, Kapp N. Intrauterine device insertion in the postpartum period: a systematic review. Eur J Contracept Reprod Health Care. 2015 Feb;20(1):4-18.
71. Switzer NJ, Dykstra MA, Gill RS, Lim S, Lester E, de Gara C, Shi X, Birch DW, Karmali S. Endoscopic versus open component separation: systematic review and meta-analysis. Surg Endosc. 2015 Apr;29(4):787-95.
72. Taglieri N, Bacchi Reggiani ML, Ghetti G, Saia F, Dall'Ara G, Gallo P, Moretti C, Palmerini T, Marrozzini C, Marzocchi A, Rapezzi C. Risk of Stroke in Patients with Stable Coronary Artery Disease Undergoing Percutaneous Coronary Intervention versus Optimal Medical Therapy: Systematic Review and Meta-Analysis of Randomized Controlled Trials. PLoS One. 2016 Jul 8;11(7):e0158769.
73. Tang Z, Yang Y, Yang Z, Meng W, Li X. Early precut sphincterotomy does not increase the risk of adverse events for patients with difficult biliary access: A systematic review of randomized clinical trials with meta-analysis and trial sequential analysis. Medicine (Baltimore). 2018 Sep;97(36):e12213.
74. Vaos G, Dimopoulou A, Gkioka E, Zavras N. Immediate surgery or conservative treatment for complicated acute appendicitis in children? A meta-analysis. J Pediatr Surg. 2019 Jul;54(7):1365-1371.
75. Wang FB, Pu YW, Zhong FY, Lv XD, Yang ZX, Xing CG. Laparoscopic permanent sigmoid stoma creation through the extraperitoneal route versus transperitoneal route. A meta-analysis of stoma-related complications. Saudi Med J. 2015 Feb;36(2):159-63.
76. Wazir U, El Hage Chehade H, Headon H, Oteifa M, Kasem A, Mokbel K. Oncological Safety of Lipofilling in Patients with Breast Cancer: A Meta-analysis and Update on Clinical Practice. Anticancer Res. 2016 Sep;36(9):4521-8.
77. Winberg H, Arnbjörnsson E, Anderberg M, Stenström P. Postoperative outcomes in distal hypospadias: a meta-analysis of the Mathieu and tubularized incised plate repair methods for development of urethrocutaneous fistula and urethral stricture. Pediatr Surg Int. 2019 Nov;35(11):1301-1308.
78. Wu Y, Mu Y, Yin L, Wang Z, Liu W, Wan H. Complications in the Management of Acute Achilles Tendon Rupture: A Systematic Review and Network Meta-analysis of 2060 Patients. Am J Sports Med. 2019 Jul;47(9):2251-2260.
79. Wusiman P, Tayie A, Ling W, Moming A. Management of Mandibular Fractures Using Locking and Nonlocking Miniplates. J Craniofac Surg. 2019 Mar/Apr;30(2):448-452.
80. Xia Y, Zhao J, Cao DS. Safety of Lipoabdominoplasty Versus Abdominoplasty: A Systematic Review and Meta-analysis. Aesthetic Plast Surg. 2019 Feb;43(1):167-174.
81. Xu R, Lian Y, Li WX. Airway Complications during and after General Anesthesia: A Comparison, Systematic Review and Meta-Analysis of Using Flexible Laryngeal Mask Airways and Endotracheal Tubes. PLoS One. 2016 Jul 14;11(7):e0158137.
82. Xu N, Yu M, Liu X, Sun C, Chen Z, Liu Z. A systematic review of complications in thoracic spine surgery for ossification of the posterior longitudinal ligament. Eur Spine J. 2017 Jul;26(7):1803-1809.
83. Yahav D, Green H, Eliakim-Raz N, Mor E, Husain S. Early double J stent removal in renal transplant patients to prevent urinary tract infection - systematic review and meta-analysis of randomized controlled trials. Eur J Clin Microbiol Infect Dis. 2018 Apr;37(4):773-778.
84. Yang Y, Ma L, Liu H, Xu M. A Meta-Analysis of the Incidence of Patient-Reported Dysphagia After Anterior Cervical Decompression and Fusion with the Zero-Profile Implant System. Dysphagia. 2016 Apr;31(2):134-45.
85. Yoon BH, Ha YC, Lee YK, Koo KH. Postoperative Deep Infection After Cemented Versus Cementless Total Hip Arthroplasty: A Meta-Analysis. J Arthroplasty. 2015 Oct;30(10):1823-7.
86. Yoon BH, Seo JG, Koo KH. Comparison of Postoperative Infection-Related Complications between Cemented and Cementless Hemiarthroplasty in Elderly Patients: A Meta-Analysis. Clin Orthop Surg. 2017 Jun;9(2):145-152.
87. Yuan Y, Peizhi Z, Xiang W, Yanhui L, Ruofei L, Shu J, Qing M. Intraoperative seizures and seizures outcome in patients undergoing awake craniotomy. J Neurosurg Sci. 2019 Jun;63(3):301-307.
88. Yuan ZZ, Yang Z, Liu Q, Liu YM. Complications following open reduction and internal fixation versus external fixation in treating unstable distal radius fractures: Grading the evidence through a meta-analysis. Orthop Traumatol Surg Res. 2018 Feb;104(1):95-103.
89. Zhao X, Wu X, Dong J, Liu Y, Zheng L, Zhang L. A Meta-analysis of Postoperative Complications of Tissue Expander/Implant Breast Reconstruction Using Acellular Dermal Matrix. Aesthetic Plast Surg. 2015 Dec;39(6):892-901.
90. Ullman AJ, Marsh N, Mihala G, Cooke M, Rickard CM. Complications of Central Venous Access Devices: A Systematic Review. Pediatrics. 2015;136(5):e1331-e1344.
91. Kirby A, Hobson RP, Burke D, Cleveland V, Ford G, West RM. Appendicectomy for suspected uncomplicated appendicitis is associated with fewer complications than conservative antibiotic management: a meta-analysis of post-intervention complications. J Infect. 2015;70(2):105-110.
92. Doleman B, Moppett IK. Is early hip fracture surgery safe for patients on clopidogrel? Systematic review, meta-analysis and meta-regression. Injury. 2015;46(6):954-962.
93. Dinh K, Limmer AM, Paravastu SCV, et al. Mortality After Paclitaxel-Coated Device Use in Dialysis Access: A Systematic Review and Meta-Analysis. J Endovasc Ther. 2019;26(5):600-612.
94. Mathew PJ, Mathew JL. Early versus late removal of the laryngeal mask airway (LMA) for general anaesthesia. Cochrane Database Syst Rev. 2015;(8):CD007082.
95. Buller M, Schulz S, Kasdan M, Wilhelmi BJ. The Incidence of Complex Regional Pain Syndrome in Simultaneous Surgical Treatment of Carpal Tunnel Syndrome and Dupuytren Contracture. Hand (N Y). 2018;13(4):391-394.
96. Gaffar R, Habib B, Filion KB, Reynier P, Eisenberg MJ. Optimal Timing of Complete Revascularization in Acute Coronary Syndrome: A Systematic Review and Meta-Analysis. J Am Heart Assoc. 2017;6(4):e005381.

**31 systematic reviews without meta-analyses**

1. Abouel-Enin S, Fraig H, Griffiths J, Latham J. Intra-pelvic migration of femoral head trial in total hip arthroplasty, a rare intra-operative complication: a systematic literature review. Musculoskelet Surg. 2016 Aug;100(2):77-81.
2. Ali AS, Benton JA, Yates JM. Risk of inferior alveolar nerve injury with coronectomy vs surgical extraction of mandibular third molars-A comparison of two techniques and review of the literature. J Oral Rehabil. 2018 Mar;45(3):250-257.
3. Badger J. Long peripheral catheters for deep arm vein venous access: A systematic review of complications. Heart Lung. 2019 May-Jun;48(3):222-225.
4. Chan A, Parent E, Narvacan K, San C, Lou E. Intraoperative image guidance compared with free-hand methods in adolescent idiopathic scoliosis posterior spinal surgery: a systematic review on screw-related complications and breach rates. Spine J. 2017 Sep;17(9):1215-1229.
5. Dawson VS, Amjad S, Fransson H. Endodontic complications in teeth with vital pulps restored with composite resins: a systematic review. Int Endod J. 2015 Jul;48(7):627-38.
6. De Lima A, Galjart B, Wisse PH, Bramer WM, van der Woude CJ. Does lower gastrointestinal endoscopy during pregnancy pose a risk for mother and child? - a systematic review. BMC Gastroenterol. 2015 Feb 12;15:15.
7. Demirbas BT, Gulluoglu BM, Aktan AO. Retained abdominal gallstones after laparoscopic cholecystectomy: a systematic review. Surg Laparosc Endosc Percutan Tech. 2015 Apr;25(2):97-9.
8. Foran T, Butcher BE, Kovacs G, Bateson D, O'Connor V. Safety of insertion of the copper IUD and LNG-IUS in nulliparous women: a systematic review. Eur J Contracept Reprod Health Care. 2018 Oct;23(5):379-386.
9. Gommers JSM, Diederen M, Wilkinson C, Turnbull D, Mol BWJ. Risk of maternal, fetal and neonatal complications associated with the use of the transcervical balloon catheter in induction of labour: A systematic review. Eur J Obstet Gynecol Reprod Biol. 2017 Nov;218:73-84.
10. Gorter RR, The SML, Gorter-Stam MAW, Eker HH, Bakx R, van der Lee JH, Heij HA. Systematic review of nonoperative versus operative treatment of uncomplicated appendicitis. J Pediatr Surg. 2017 Aug;52(8):1219-1227.
11. Iluyomade A, Olowoyeye A, Fadahunsi O, Thomas L, Libend CN, Ragunathan K, Fenster J, Vignesh S. Interference with daily activities and major adverse events during esophageal pH monitoring with bravo wireless capsule versus conventional intranasal catheter: a systematic review of randomized controlled trials. Dis Esophagus. 2017 Feb 1;30(3):1-9.
12. Kamatham R, Avisa P, Vinnakota DN, Nuvvula S. Adverse Effects of Implants in Children and Adolescents: A Systematic Review. J Clin Pediatr Dent. 2019;43(2):69-77.
13. Kohn JR, Shamshirsaz AA, Popek E, Guan X, Belfort MA, Fox KA. Pregnancy after endometrial ablation: a systematic review. BJOG. 2018 Jan;125(1):43-53.
14. Liguori G, Dobrinja C, Pavan N, de Manzini N, Bucci S, Palmisano S, Trombetta C. Iatrogenic ureteral injury during laparoscopic colectomy: incidence and prevention A current literature review. Ann Ital Chir. 2016;87:446-455.
15. Liu YM, Xie P. The Safety of Orthokeratology--A Systematic Review. Eye Contact Lens. 2016 Jan;42(1):35-42.
16. Malik T, Lee MJ, Harikrishnan AB. The incidence of stoma related morbidity - a systematic review of randomised controlled trials. Ann R Coll Surg Engl. 2018 Sep;100(7):501-508.
17. Martins OFM, Chaves Junior CM, Rossi RRP, Cunali PA, Dal-Fabbro C, Bittencourt L. Side effects of mandibular advancement splints for the treatment of snoring and obstructive sleep apnea: a systematic review. Dental Press J Orthod. 2018 Aug 1;23(4):45-54.
18. Mohamad S, Khan I, Hey SY, Hussain SS. A systematic review on skin complications of bone-anchored hearing aids in relation to surgical techniques. Eur Arch Otorhinolaryngol. 2016 Mar;273(3):559-65.
19. Molinero-Mourelle P, Baca-Gonzalez L, Gao B, Saez-Alcaide LM, Helm A, Lopez-Quiles J. Surgical complications in zygomatic implants: A systematic review. Med Oral Patol Oral Cir Bucal. 2016 Nov 1;21(6):e751-e757.
20. Muskus M, Rojas J, Gutiérrez C, Guio J, Bonilla G, Llinás A. Bilateral Hip Arthroplasty: When Is It Safe to Operate the Second Hip? A Systematic Review. Biomed Res Int. 2018 Feb 28;2018:3150349.
21. Petrillo M, Nero C, Carbone V, Bruno M, Scambia G, Fagotti A. Systematic Review of Cytoreductive Surgery and Bevacizumab-Containing Chemotherapy in Advanced Ovarian Cancer: Focus on Safety. Ann Surg Oncol. 2018 Jan;25(1):247-254.
22. Scerrino G, Melfa G, Raspanti C, Rotolo G, Salamone G, Licari L, Fontana T, Tutino R, Porrello C, Gulotta G, Cocorullo G. Minimally Invasive Video-Assisted Thyroidectomy: Analysis of Complications From a Systematic Review. Surg Innov. 2019 Jun;26(3):381-387.
23. Shah A, Kay J, Memon M, Coughlin RP, Simunovic N, Nho SJ, Ayeni OR. What Makes Suture Anchor Use Safe in Hip Arthroscopy? A Systematic Review of Techniques and Safety Profile. Arthroscopy. 2019 Apr;35(4):1280-1293.e1.
24. Shen X, Zhang X, Bi J, Yin K. Long-term complications requiring reoperations after laparoscopic adjustable gastric banding: a systematic review. Surg Obes Relat Dis. 2015 Jul-Aug;11(4):956-64.
25. Soro MP, Denys A, de Rham M, Baud D. Short & long term adverse outcomes after arterial embolisation for the treatment of postpartum haemorrhage: a systematic review. Eur Radiol. 2017 Feb;27(2):749-762.
26. Sorrentino R, Di Mauro MI, Ferrari M, Leone R, Zarone F. Complications of endodontically treated teeth restored with fiber posts and single crowns or fixed dental prostheses-a systematic review. Clin Oral Investig. 2016 Sep;20(7):1449-57.
27. Stacchi C, Andolsek F, Berton F, Perinetti G, Navarra CO, Di Lenarda R. Intraoperative Complications During Sinus Floor Elevation with Lateral Approach: A Systematic Review. Int J Oral Maxillofac Implants. 2017 May/Jun;32(3):e107-e118.
28. van Esch BF, Stegeman I, Smit AL. Comparison of laryngeal mask airway vs tracheal intubation: a systematic review on airway complications. J Clin Anesth. 2017 Feb;36:142-150.
29. Waked K, Colle J, Doornaert M, Cocquyt V, Blondeel P. Systematic review: The oncological safety of adipose fat transfer after breast cancer surgery. Breast. 2017 Feb;31:128-136.
30. Willner D, Spennati V, Stohl S, Tosti G, Aloisio S, Bilotta F. Spine Surgery and Blood Loss: Systematic Review of Clinical Evidence. Anesth Analg. 2016 Nov;123(5):1307-1315.
31. Yamaguchi K, Lonic D, Lo LJ. Complications following orthognathic surgery for patients with cleft lip/palate: A systematic review. J Formos Med Assoc. 2016 Apr;115(4):269-77.
